# The Effect of Anchoring Sutures on Medicinal Leech Mortality

**Published:** 2009-07-21

**Authors:** Victor J. Davila, Ian C. Hoppe, Rocco Landi, Frank S. Ciminello

**Affiliations:** New Jersey Medical School, University of Medicine & Dentistry of New Jersey, Newark.

## Abstract

**Objective:** The implementation of leech therapy for surgical flaps is not always logistically easy or comfortable for patients or healthcare providers. We examine different methods of placing sutures in the medicinal leech, *Hirudo medicinalis*, to make the implementation of leech therapy easier. **Methods:** Sixteen leeches were randomly divided into 3 groups: a control group, a deep anchoring suture group, and a superficial anchoring suture group. The leeches were observed to determine if either of these methods had an adverse effect on survival compared with the control group. **Results:** No difference in survival time was observed across the different groups. **Conclusion:** The placement of anchoring sutures in leeches can ease the implementation of leech therapy by allowing for greater control of the leeches and thus increased patient comfort.

The medicinal leech, *Hirudo medicinalis*, is used by plastic and reconstructive surgeons in the postoperative period to relieve surgical site venous congestion, and this has been shown to improve surgical outcomes.^1^ However, 2 practical issues limit their use: (1) leech migration often causes patient distress and (2) medical personnel often have difficulties with leech placement and retrieval. Reports of leech migration from the surgical site after feeding has prompted the examination of leech-anchoring methods. A recent study examined a method of anchoring medicinal leeches with a simple suture to limit their migration away from the surgical site and to aid in retrieval when feeding has ended.^2^ The purpose of the present study was to examine leech mortality when the use of different types of anchoring sutures (superficial vs. deep sutures) were placed through the leech.

## METHODS

**Figure 1:**
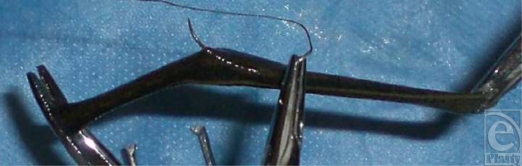
Superficial suture placement.

Sixteen leeches were available for analysis; these were randomly divided into 3 groups from a common container: control (*n* = 4), deep suture placement (*n* = 6), and superficial suture placement (*n* = 6). Leeches in the deep suture placement group had a suture placed through the middle of the body that exited on the opposite side of the insertion point. Leeches in the superficial placement group also had a suture placed in the middle of the body, but the suture needle penetrated only the outer surface of the leech to a depth of approximately 1 mm (Fig 1). Black braided 4-0 silk sutures were used and blunt forceps were applied at both ends of each leech for control while placing sutures. All sutures were secured to the leech with nontightened knots (air knots).

All leeches were subjected to the same environmental conditions. Leeches were individually placed in numbered, clear plastic containers with approximately 24 mL of water distributed from a common container. The plastic containers were covered with clear plastic wrap and secured in place with rubber bands and tape. Ventilation holes were created and the plastic containers were stored at room temperature. Leeches were examined twice a day for 7 days and assessed for leech mortality and movement in their individual containers.

## RESULTS

Over the course of the 7-day experiment, all leeches survived and exhibited similar activity levels in all 3 groups. On day 3, one of the leeches in the superficial suture group was observed to no longer be anchored to the suture because the suture was noted to have torn through the outer layer of the leech body. However, this leech continued to exhibit the same degree of movement as the rest of the leeches in both groups. Thus, the presence or absence of an anchoring suture did not seem to affect leech mobility and did not have an immediate impact on leech survival.

## CONCLUSION

This experiment was designed to examine the immediate impact of suture placement on leech survival. The results demonstrated no differences in leech survival or mobility between the suture groups (deep or superficial) or when compared with the control group.

## DISCUSSION

For thousands of years, the medicinal leech, *Hirudo medicinalis*, has been used to treat a wide array of human ailments. The practice of bloodletting in general was used by Egyptian, Greek, and Roman cultures.^3^ Bloodletting was accomplished through venesection or application of leeches and was thought to restore balance between the 4 humors proposed by Hippocrates. Leeches were used to treat various conditions including infectious diseases, inflammation, and even as a cure for bleeding itself. Use was commonplace until the late 19th century when overcollection of leeches threatened wild populations. During this time, the medical community also began to discredit their use.

Modern medicine has reintroduced the use of medicinal leeches primarily in the realm of reconstructive surgery. During surgical construction of local and free flaps or replanted digits, venous outflow is often compromised, causing venous congestion of the surgical site which can compromise tissue viability. Leeches have been shown to alleviate local venous congestion in the postoperative period, thus allowing the body time to reestablish venous outflow.^4^ This is accomplished through 2 main mechanisms: direct removal of blood and introduction of leech saliva, which contains numerous chemicals including factors that provide anticoagulation, platelet inactivation, vasodilation, and local anesthesia. Hirudin, the most potent natural inhibitor of thrombin, is the primary anticoagulant that allows the leech to feed.^5^ The vasodilatory effects of leech saliva also contribute to increased blood flow during feeding and delayed clotting of the attachment site after the leech has fallen off its host. *Hirudo medicinalis* can remove up to 9 times its own body weight (5–10 mL) of blood from a host over a 20- to 30-minute period, and each feed can provide the leech with nutrients for several months.^3^

Postoperative use of medicinal leeches usually lasts several days, with several feeding sessions per day. For each feeding session, 1 or more leeches can be used depending on the size and severity of the congested area. Often the area of concern is not easily visualized or accessible by the patient. In these situations, the leeches often migrate to other areas of the patient body or even within the hospital room. Historically, leech migration was an issue with writings of “wandering leeches” being swallowed by patients or finding their way into other body orifices.^3^ More recently, reports of leeches inching their way across hospital beds or patient rooms are not uncommon. Recently, Granzow et al^2^ described the use of an anchoring suture to immobilize the leeches and prevent movement after feeding sessions. This group described the use of 4-0 or 5-0 sutures as “passed through the leech approximately halfway along the length of its body, 2 to 3 mm from its side.” These researchers then secured the suture through the leech to a stack of surgical gauze. In addition, they noted that “[t]he application of the suture in no way seems to limit the ability of the leech to feed or move within the confines of the length of the suture.” Although the authors stated that the leeches continued normal feeding and movement after the placement of an anchoring suture, the issue of leech viability after suture placement was not addressed.

The standard method for applying a leech involves loading one into an empty syringe without a plunger and pressing the open end over the desired treatment area^4^; this can be a time-consuming activity for healthcare providers. The results of the present study suggest that it is possible to place a suture through many leeches at one time, thus making it easier to retrieve a single leech from a container and lessen the need for cumbersome forceps or empty syringes while handling them. The same anchoring suture can then be used to confine the leech to the surgical site during and after feeding. The present study supports the conclusion that a simple suture through the body of a leech does not alter short-term viability. In addition, the anchoring suture can aid in leech placement for the initiation of feeding, prevent leech migration during and after feeding, and simplify postfeeding retrieval of the leech.

The primary limitations of this study are the lack of data surrounding leech feeding habits and whether an anchoring suture affects the quantity of blood removed. This information would be useful if a difference can be found because it would impact the number of leeches needed to treat a particular area of venous congestion and the time interval between feeds.

As leeches continue to prove their usefulness in postsurgical management, this method can save time and increase the likelihood of a positive outcome by preventing venous congestion. Even outside the realm of surgery, leeches are becoming revered for their medicinal qualities. Their use has even been indicated in the treatment of osteoarthritis.^6^,^7^ Leeches are a valuable tool to the physician, and the present study shows that some of the limitations of leech therapy can be minimized by anchoring the leeches with a suture.
